# Supplementary material for: Dynamic transcriptomic profiles of zebrafish gills in response to zinc supplementation
Source: BMC Genomics. 2010 Oct 11;11:553. doi: 10.1186/1471-2164-11-553 (PMC3091702; doi:10.1186/1471-2164-11-553)
Supplement: Additional file 2 — Interactive Direct Interaction Network representing the molecular interactions between zinc, copper, iron, calcium and proteins encoded by transcripts changed by zinc supplementation. Mini web-site containing index.html and hyperlinked pages in subdirectory describing a Direct Interaction Network automatically generated based on curated interactions contained within the proprietary PathwayArchitect database. Ovals represent proteins and the circles symbolize metal ions. Objects are coloured by their abundance in zebrafish at the time-point they were significantly different from the control is a scale from -4 fold (dark green) to +4 fold (dark red). Where significant differences were found at more than one time-point, the colour overlay shows expression at the first instance. Dark blue squares denote 'binding', and light blue squares 'expression'; green squares stand for 'regulation', green diamonds for 'metabolism', and green circles for 'promoter binding'. Arrow heads indicate directionality of the interaction where annotated. All nodes and edges can be further interrogated by selecting the relative area of the image. [file 1471-2164-11-553-S2.zip › PathwayArchitect Zn xs DIN/573449.html]

# REGULATION:

|  |  |
| --- | --- |
| Type | REGULATION |
| Effect | None |


---

|  |  |
| --- | --- |
| Score | 0 |


---

|  |  |
| --- | --- |
| Reference Count | 11 |


---

|  |  |
| --- | --- |
| Mechanism | Unknown |


---

|  |  |
| --- | --- |
| Reference:0 || Sentence | "This paper examines the development of iron (Fe) deficiency and its possible interactions with trace elements such as zinc (Zn) and copper (Cu) by investigating iron-deficient and control rats." |
| PMID | 9718478 |
| Year | 1998 |
| Species | Rat |
| Journal | Lab Anim |
| RefScore | 1 |
| Source | PArchNLP |
  |
|  |  |


---

|  |  |
| --- | --- |
 Reference:1 || Sentence | "The main outcome measures were as follows: (i) percentages and absolute counts of lymphocyte subsets, (ii) association between biochemical indices of nutritional status (ferritin, iron and zinc) and peripheral blood lymphocyte subsets, (iii) total mortality during a 22-month period in relation to baseline lymphocyte subset counts." |
| PMID | 15003923 |
| Year | 2004 |
| Species | Human |
| Journal | Asia Pac J Clin Nutr |
| RefScore | 1 |
| Source | PArchNLP |
  ||  |  |


---

|  |  |
| --- | --- |
 Reference:2 || Sentence | "The amount of Zn, Fe, Cu, Mg and Ca in rat liver samples was analysed by atomic-absorption spectrophotometry and the association of Zn, Fe and Mg to cytosol proteins was studied by ultrafiltration." |
| PMID | 755325 |
| Year | 1978 |
| Species | Rat |
| Journal | Acta Biochim Biophys Acad Sci Hung |
| RefScore | 1 |
| Source | PArchNLP |
  ||  |  |


---

|  |  |
| --- | --- |
 Reference:3 || Sentence | We examined the association of protein, iron, zinc, vitamin B-12, and folic acid with tests of acquired immunity in healthy older women (76.7 +/- 7.0 y; n = 130). |
| Year | 2005 |
| PMID | 16251624 |
| Species | Human |
| Journal | J Nutr |
| RefScore | 0 |
| Source | PArchNLP |
  ||  |  |


---

|  |  |
| --- | --- |
 Reference:4 || Sentence | On the other hand, Al, Fe, Mn, Cu, and Zn concentrations in hippocampus were also determined. |
| PMID | 15589378 |
| Year | 2005 |
| Species | Rat |
| Journal | Free Radic Biol Med |
| RefScore | 2 |
| Source | PArchNLP |
  ||  |  |


---

|  |  |
| --- | --- |
 Reference:5 || Sentence | In the current study, dietary intakes of Fe and Zn fractions (total Fe, non-haem Fe, haem Fe, meat Fe, total Zn) and dietary components that influence Fe and Zn absorption (vitamin C, phytate, Ca, grams of meat/fish/poultry, black tea equivalents, phytate:Zn molar ratio) were assessed. |
| PMID | 15877881 |
| Year | 2005 |
| Species | Human |
| Journal | Br J Nutr |
| RefScore | 2 |
| Source | PArchNLP |
  ||  |  |


---

|  |  |
| --- | --- |
 Reference:6 || Sentence | We reviewed randomized trials that assessed the effects of iron and zinc supplementation on iron and zinc status. |
| PMID | 16002793 |
| Year | 2005 |
| Species | Human |
| Journal | Am J Clin Nutr |
| RefScore | 2 |
| Source | PArchNLP |
  ||  |  |


---

|  |  |
| --- | --- |
 Reference:7 || Sentence | Zinc and copper status are modified by iron manipulation. |
| PMID | 16038040 |
| Year | 2005 |
| Species | Rat |
| Journal | World J Gastroenterol |
| RefScore | 2 |
| Source | PArchNLP |
  ||  |  |


---

|  |  |
| --- | --- |
 Reference:8 || Sentence | On the other hand, Al, Fe, Mn, Cu and Zn concentrations were determined in cortex and cerebellum of rats. |
| PMID | 16098089 |
| Year | 2005 |
| Species | Rat |
| Journal | J Pineal Res |
| RefScore | 1 |
| Source | PArchNLP |
  ||  |  |


---

|  |  |
| --- | --- |
 Reference:9 || Sentence | Metal analysis showed that the recombinant enzyme contained a mixture of iron, nickel, and zinc, with increased iron content associated with increased catalytic activity. |
| PMID | 16492780 |
| Year | 2006 |
| Species | Mouse |
|  | Human |
| Journal | Proc Natl Acad Sci U S A |
| RefScore | 1 |
| Source | PArchNLP |
  ||  |  |


---

|  |  |
| --- | --- |
 Reference:10 || Sentence | CONCLUSIONS: Combined iron-zinc supplementation had a positive effect on iron and zinc status in infants. |
| PMID | 16306925 |
| Year | 2006 |
| Species | Human |
| Journal | Eur J Clin Nutr |
| RefScore | 1 |
| Source | PArchNLP |
  |


---

|  |  |
| --- | --- |
